# Targeting cellular senescence in progenitor cells as a strategy to enhance bone regeneration by cell therapies: a systematic review of pre-clinical investigations

**DOI:** 10.1186/s13287-025-04767-8

**Published:** 2025-11-28

**Authors:** Mayu Morita, Eshan B. Damle, Issei Shinohara, Masatoshi Murayama, Yosuke Susuki, Qi Gao, Chao Ma, Simon Kwoon-Ho Chow, Stuart Barry Goodman

**Affiliations:** 1 Department of Orthopaedic Surgery, School of Medicine, Stanford University, Palo Alto, CA USA; 2 Department of Bioengineering, Stanford University, Palo Alto, CA USA; 3 Department of Dentistry and Oral Surgery, Keio University School of Medicine, Tokyo, Japan; 4 School of Medicine, Stanford University, Palo Alto, CA USA

**Keywords:** Bone regeneration, Cellular senescence, Senescence marker, ROS, p53, p21, p16

## Abstract

**Background:**

With the global population aging, optimizing bone regeneration is becoming increasingly important for enhancing the quality of life among elderly individuals. Progenitor cell-based therapies, such as mesenchymal stromal cells and induced pluripotent stem cells for bone regeneration have shown challenges due to cellular senescence and the control of the differentiation processes remain significant hurdles. In particular, elevated expression of senescence markers may play a pivotal role in limiting bone regeneration. This systematic review examines how these senescence markers influence the efficacy of progenitor cell therapies and whether targeting them could improve outcomes.

**Methods:**

We conducted a systematic literature review following the PRISMA guidelines, using the PubMed, Web of Science, Embase and Scopus with the algorithm of “bone regeneration AND senescence AND marker”. Data synthesis focused on human cell sources and specifically examined senescence markers related to bone regeneration.

**Results:**

Studies using human cells were discussed in 101 papers. Based on our inclusion and exclusion criteria, 13 papers remained for our review on senescence markers in human cells within the context of bone regeneration and senescence, with and without interventional strategies. More than half of the cell sources in current aging-related studies are derived from bone marrow. Markers of aging relevant to bone regeneration include changes in cell size and morphology, increased levels of β-galactosidase (β-Gal) and Reactive Oxygen Species (ROS), and the presence of a senescence-associated secretory phenotype (SASP). Additionally, distinct senescence markers such as p16Ink4a, p21, and p53, and mitochondrial dysfunction were associated with reduced osteogenic potential and impaired regenerative capacity.

**Conclusion:**

Bone marrow is the most common source of cells for studies of senescence. Cellular senescence characterized by elevated expression of specific markers was consistently shown to be negatively associated with osteogenic capacity and regenerative outcomes. The most common strategies to rejuvenate senescent cells include targeting of senescence markers and oxidative stress. Among these, modulation of p53, p21, and p16 signaling pathways has been highlighted as a potential therapeutic approach for mitigating cell senescence in bone-related conditions.

## Introduction

Bone regeneration therapy is becoming increasingly important as the world’s population ages with increasing life expectancies. According to United Nations’ “World Population Prospects 2022”, by 2050, the global population of 65 years old or older will more than double, reaching over 1.5 billion globally [1]. Maintaining bone health in an aging society is directly linked to improving quality of life. In elderly individuals, the decrease in bone density and the increased risk of fractures compromise their independence, making bone regeneration therapy a critical, yet unmet clinical need [2].

In orthopaedics and dentistry, progress in bone regeneration therapy enables safer and more effective treatments for elderly patients and those with impaired healing potential, leading to improvements in functional outcomes such as ambulation, autonomy, and masticatory efficiency, ultimately enhancing patients’ overall quality of life [3–5].

Recently, significant attention has been given to the clinical application of mesenchymal stromal cells (MSCs) in the field of bone regeneration [6, 7]. These self-renewing multipotent cells can differentiate into bone [8], cartilage [9], and adipose tissue [10], as well as other cell types under in vitro conditions. Interestingly, recent findings have highlighted that MSCs transplanted in vivo promoted bone regeneration primarily through paracrine effects by secreting immunomodulatory factors contributing to the polarization of M0 macrophages to the M2 phenotype [11, 12]. The advantages of using MSCs include self-renewal, multipotency, and immunomodulatory capabilities, thus reducing the risk of immune rejection with enhanced safety compared to other treatments [13]. However, age-related decline in MSC function and differentiation capacity remain major challenges.

Cellular senescence (or aging) presents a significant obstacle in bone regeneration therapy. Senescent cells secrete substances broadly defined as senescence-associated secretory phenotype (SASP). It may contain soluble signaling factors such as interleukins, chemokines, growth factors, proteases, insoluble proteins or ECM components. These substances negatively affect surrounding cells, causing inflammation and tissue dysfunction, thereby impacting the bone regeneration process. Senescence markers such as p16Ink4a, p21 and p53 that are cell cycle inhibitors, are also known to play crucial roles in cellular senescence [14–16]. Expression of these factors are generally induced in response to DNA damage and stress, thus leading to cell cycle arrest and senescence [17].

Moreover, the accumulation of reactive oxygen species (ROS) and mitochondrial dysfunction are involved in the mechanisms of cellular senescence. ROS are naturally generated within cells. However, excessive accumulation of ROS causes damage to DNA, proteins, and lipids [18]. When mitochondrial function declines, energy production becomes inefficient, and ROS production increases, creating a vicious dysregulated cycle. These factors all contribute to the progression of cellular senescence that eventually impairs tissue regenerative capacity [19].

The aim of this systematic review is to survey existing literature to address the question whether cellular senescence signified by associated senescence markers such as p16, p21, p53, ROS, and SASP, play a role in the efficacy of progenitor cell therapies specifically on bone regeneration; and if targeting cellular senescence would improve treatment efficacies.

## Materials and methods

### Search strategy

A systematic literature search was conducted in PubMed, Web of Science, Embase and Scopus with the keywords “bone regeneration”, “senescence” and “marker” (Last access on 28 July 2025). We combined the keywords as “bone regeneration AND senescence AND marker” and followed Preferred Reporting Items of Systematic reviews and Meta-analyses (PRISMA) guidelines. Data synthesis focused on human cell sources and specifically examined senescence markers related to bone regeneration.

### Search criteria

The inclusion criteria were: (1) studies on bone regeneration and senescence markers, (2) human cell sources (i.e. examine senescence in vitro using human cells or in vivo using human cells administered to animals), (3) bone-related outcomes (including alveolar bone), (4) full-text literature in English. The exclusion criteria were: (1) review articles, (2) conference abstracts, (3) non-senescence related, (4) non-bone related, (5) without full-text access (i.e. publications that were neither open access nor accessible through the full range of resources available via our university resources); and (6) animal cell sources.

### Study selection

Two independent reviewers conducted the study selection process. Initially, they excluded clearly irrelevant papers based on titles and abstracts. The remaining articles that seemed potentially relevant were then assessed according to the specified inclusion and exclusion criteria. If disagreements arose between the two reviewers, they engaged in discussions to reach a mutual agreement.

### Data extraction

Reviewers extracted data from the included studies, focusing on human cell sources and various senescence markers in the context of bone regeneration. These markers included (a) Senescence-Associated β-Galactosidase (SA-β-Gal), (b) P16Ink4a, (c) p21, (d) p53, (e) Reactive Oxygen Species (ROS), (f) Senescence-Associated Secretory Phenotype (SASP), and (g) mitochondrial dysfunction, and collected key findings related to bone regeneration. Cartilage regeneration was excluded from this review as the focus was specifically on MSC osteogenic differentiation.

### Data analysis

The studies related to bone regeneration and senescence markers are diverse and vary in design, methodology and quantification methods, making a meta-analysis not feasible. Therefore, a systematic review was conducted to evaluate the impact of specific senescence markers on bone regeneration from a broad perspective and to understand overall trends and research limitations.

## Results

### Search results

**Fig. 1:**
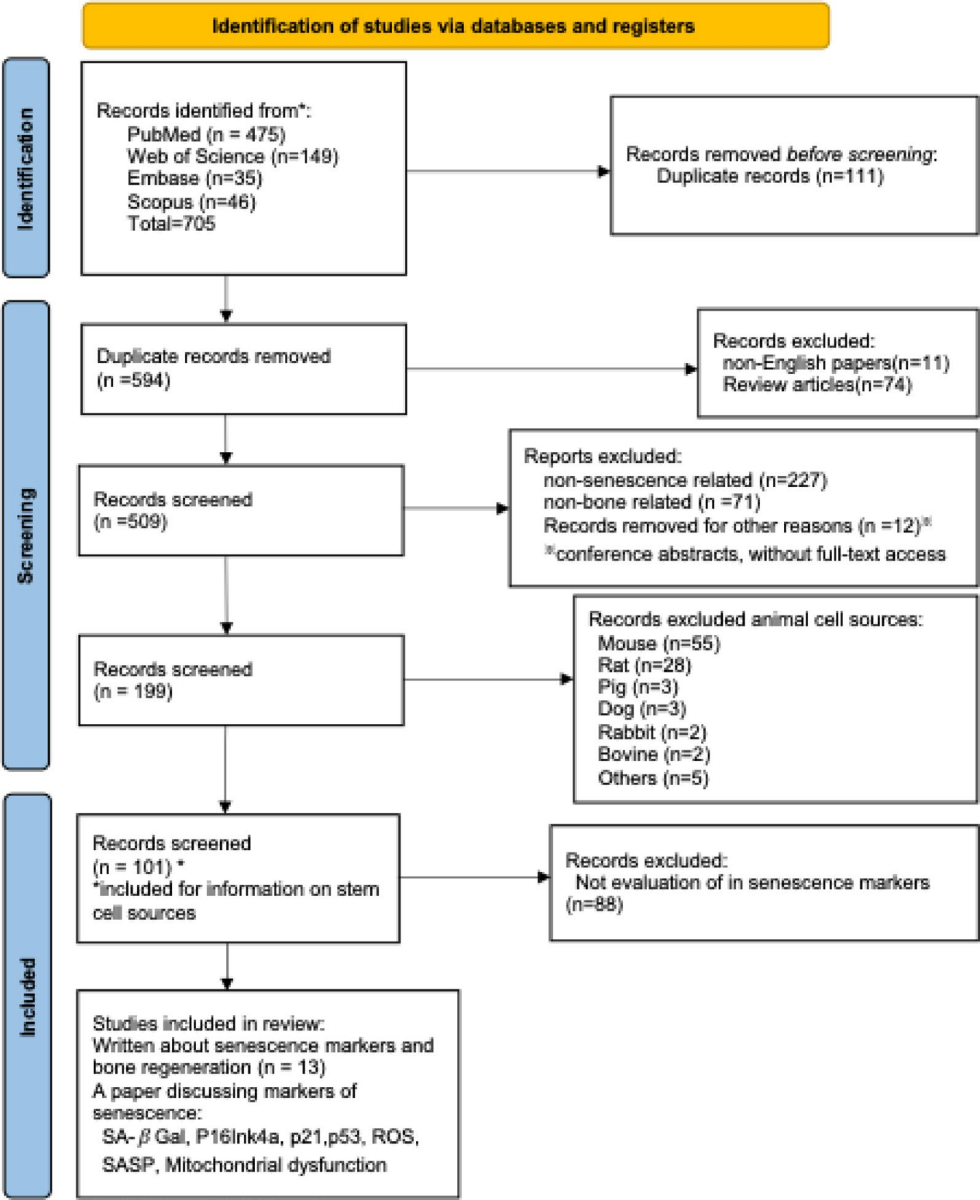
PRISMA flowchart using the key words as “bone regeneration AND senescence AND marker”

A total of 705 publications were retrieved from PubMed (475 papers), Web of Science (149 papers), Embase (35 papers) and Scopus (46 papers). After removing 111 duplicates, 594 publications remained. Titles and abstracts were screened, excluding 310 studies unrelated to bone regeneration and senescence. Studies were excluded for the following reasons: not related to cellular senescence (*n* = 227), not focused on bone regeneration, including those focused on cartilage regeneration (*n* = 71), or removed for other reasons (*n* = 12) such as being conference abstracts or no full-text access. After excluding experiments using animal cells (*n* = 98), only studies involving human cells were retained. This left 101 papers for a full-text eligibility check. Papers in which there was no evaluation of senescence markers were excluded (*n* = 88). Ultimately, 13 studies met the inclusion and exclusion criteria and were included in the systematic review. Figure 1 shows the flow diagram summarizing the selection process.

### Origins of progenitor cells

**Fig. 2:**
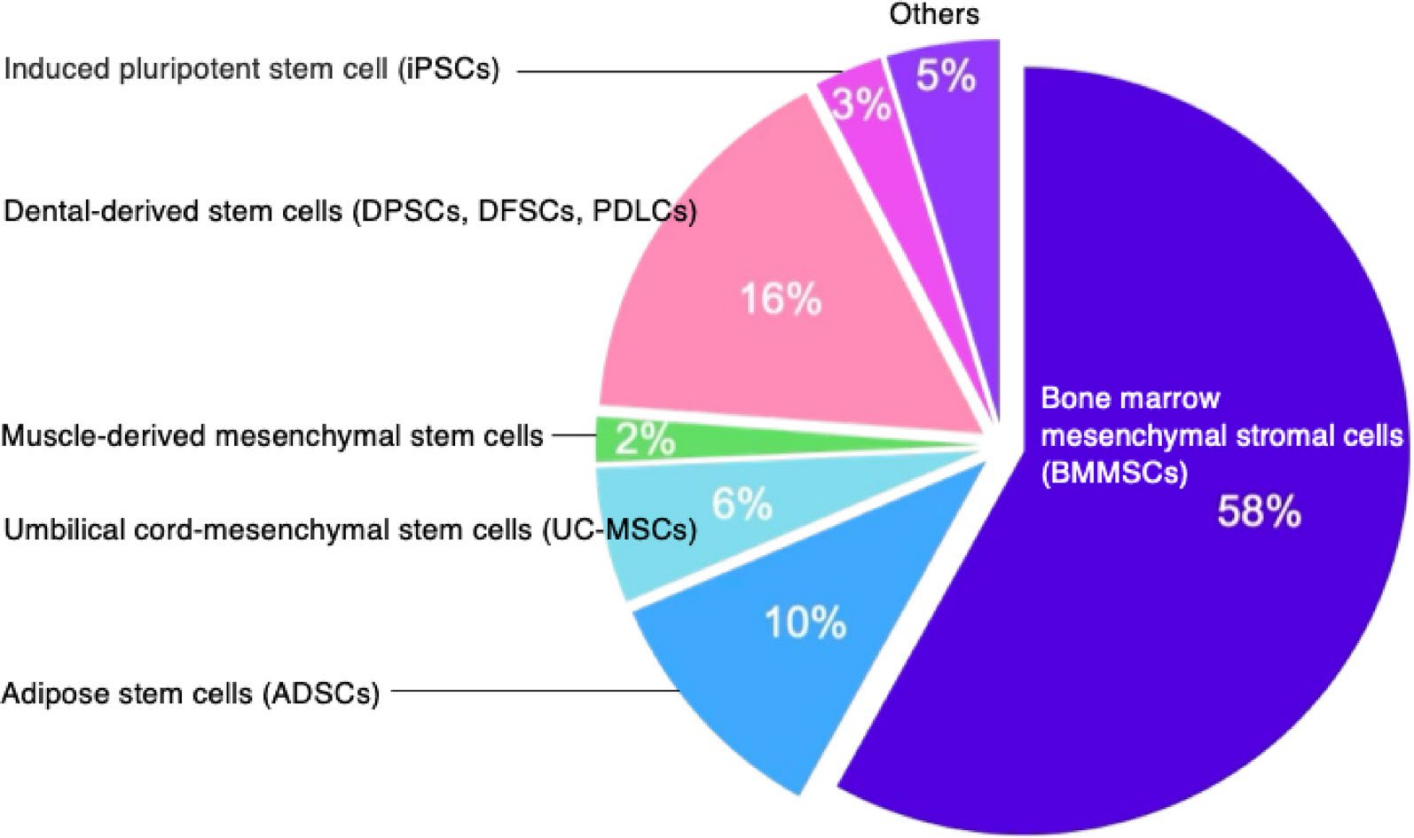
Proportion of cell sources used as an intervention strategy in the 101 studies

Stem cell transplantation is a promising strategy in bone regeneration therapy. Choices of cell sources proposed for bone regenerative therapy have been diverse. Relevant cells for regenerative therapy can now be harvested from many different tissues [20]. A large percentage of cells are sourced from the musculoskeletal system. However, due to the invasiveness of harvesting bone marrow, other tissue sources are being actively explored (see Table 1). For example, cells from dental sources, adipose tissue, and umbilical cords are being considered, largely due to factors such as ease of biopsy and availability. Figure 2 summarizes the origins of cells used in 101 papers included in this review, focusing on bone regeneration and senescence.

**Table 1:** Commonly used locations and abbreviations of various types of cells used for research in regenerative cell therapy

	Abbreviation	
Primary cell-derived	hBM-MSC	Human bone marrow mesenchymal stromal cells
	ADSC	Adipose stem cells
	UC-MSC	Umbilical cord-MSC
	Muscle-derived MSC	
Dental and periodontal tissue-derived	DPSCs	Dental pulp stem cells
	PDLCs	Periodontal ligament cells
	DFSCs	Dental follicle stem cells
	hPDLF	Human periodontal ligament fibroblasts
Fetal tissue and amniotic fluid-derived	hAFSCs	Human amniotic fluid stem cells
	hfSDSCs	Human fetal synovium-derived stem cells
Bone and bone-related tissue-derived	Cranial periosteal cells	
	BMA	Bone marrow aspirate
	HAOBs	Human osteoblasts isolated from aged alveolar bone
Stem cell-derived	iPSCs	Induced pluripotent stem cells
	Embryonic stem-derived cells	
	iMSCs	iPS-derived MSCs

#### Primary cell-derived

In these studies of bone regeneration, one prominent source was human bone marrow mesenchymal stromal cells (hBMMSCs)that accounted for over half of the 101 papers. Since harvesting bone marrow is invasive, various alternative cell sources are being considered. The next most common cell source is adipose stem cells (ADSCs). ADSCs have gained attention due to their arguably less invasive harvesting procedure and the abundant quantity that can be obtained from adipose tissue.

Other sources of MSCs include Umbilical Cord-MSCs (UC-MSCs) and muscle-derived MSCs, which offer alternative tissues for stem cell extraction. The umbilical cord tissue, or matrix (Wharton jelly-UCT), serves as a valuable source of MSCs, offering several advantages over other sources due to its non-invasive collection process and regarded as “medical waste”. Muscle-derived MSCs are being studied to determine whether they are affected by conditions like osteoarthritis (OA) or osteoporosis (OP), as bone marrow-derived MSCs that are often used as a therapeutic source may be influenced by these conditions.

#### Dental and periodontal tissue-derived

Dental tissues exhibit a variety of advantageous properties for cell-based therapies due to their ease of accessibility, minimally invasive collection, and abundant supply of stem cells. For instance, human dental pulp stem cells (DPSCs) and human periodontal ligament cells (PDLCs), dental follicle stem cells (DFSCs) and human periodontal ligament fibroblasts (hPDLF) have been investigated for their potential in dental and periodontal regeneration including alveolar bone.

#### Bone and bone-related tissue-derived

Human osteoblasts isolated from aged alveolar bone (HAOBs) are also being considered as a valuable cell source for investigation of osteogenesis and the senescence processes. Additionally, human primary cranial periosteal cells and Bone marrow aspirate (BMA) have been considered for cranial bone regeneration.

#### Fetal tissue and amniotic fluid-derived

In addition, human amniotic fluid stem cells (hAFSCs) and human fetal synovium-derived stem cells (hfSDSCs) are also being studied as cell sources for bone regeneration research.

#### Stem cell-derived

Human induced pluripotent stem cells (iPSCs) and embryonic stem-derived cells (such as 1C6) are employed to generate various cell types for regenerative medicine. Lian et al. utilized three human iPSC lines to generate MSCs. Two of the cell lines were derived from fibroblasts, while the third was derived from human foreskin. Spitzhorn et al. used mesenchymal stem cells, isolated from the bone marrow of a 74-year-old female donor, for the generation of iPSCs and iMSCs. The iPSCs in a third study were derived from a dermal fibroblast cell line and a bone marrow cell line.

These diverse cell sources highlight the complexity and promise of stem cell research in developing effective bone regeneration therapies.

### Characteristics of the study

Of the 13 studies included in this systematic review, all studies utilized cells from human sources, with the investigation on cellular senescence evaluated by the expression of senescence markers. Thirteen papers focused on bone regeneration, three of which specifically on the alveolar bone. The characteristics of the 13 included studies are summarized in Table 2.

**Table 2:** Characteristics of studies included in this systematic review. Abbrev: SAHF: senescence-associated heterochromatin foci, ALP: alkaline Phosphatase, ARS: Alizarin red Staining, ORO: oil red O staining, DOP, NAC-treated, PA-treated, microct: micro-computed tomography, ROS: reactive oxygen species, NS: not-specified

Studies	Cell source	Age, Gender Source	Sample size	Intervention	Senescence Markers	Functional outcome (in vitro)	Functional outcomes (in vivo) (progenitor cell source/ application)
Heo et al. [26]	Bone Marrow-Derived MSCs (BMMSCs)	NS, NS Bone marrow	*n* = 3	The role of APE1/Ref-1in senescence induced by oxidative stress from H₂O₂	SA-β-gal p53, p21 ROS	mRNA (*Ref-1*) Protein (Ref-1)	-
Choi et al. [32]	Dental pulp stem cells (DPSCs)	NS, NS Teeth	NS	Treatment with the LMWP-system for superoxide dismutase 1 (SOD1) under oxidative stress induced by H₂O₂	p53, p21	mRNA (*DSPP*,* COL1*,* ALP*,* OPN*,* RUNX2*,* MAX1*,* MAX2*) Protein (MAX1, MAX2) ALP, ARS	-
Chen et al. [31]	Bone Marrow-Derived MSCs (BMMSCs)	-, - Cyagen Biosciences Inc.	-	Senescence Responses to Mechanical Stimulation	ROS	mRNA (*BGLAP*,* RUNX2*,* SP7*) ALP, ARS	-
Marycz et al. [41]	Adipose-Derived Mesenchymal Stromal Stem Cells (ADSCs)	20–33, Male and female Adipose tissue	NS	The antioxidant effects of Nanocrystalline Hydroxyapatite (nHAp) Loaded with Resveratrol (RES) on cellular senescence	p53, p21 ROS Mitochondrial function	mRNA (*Mnf-1*,* Fis*,* Beclin*,* Prrkin*,* Bax*,* Bcl-2*) microRNA (miR-17, miR-145, miR-223, miR-320) MitoRed staining	-
Liu et al. [27]	Adipose-derived stem cells (ADSCs)	-, - ScienCell Research Laboratories	-	The effects of BMP antagonist, GREM1 on senescence	SA-β-gal p53, p16	mRNA (*OCN*,* OPN*,* DSPP*,* DMP1*,* BSP*,* OSX*,* Runx2*,* BMP2*,* BMP4*,* BMP5*,* BMP6*,* BMP7*,* BMP9*) ALP, ARS	-
Gresham et al. [24]	MSCs, Bone Marrow Derived Cells (BMDCs)	-, - RoosterBio Inc.	-	The effects of fisetin on senescence induced by H₂O₂, etoposide, and irradiation	SA-β-gal p21, p16 SAHF (H2AX, γH2AX) SASP (IL-6, IL-1β)	ALP, ARS alamarBlue	-
Meng et al. [28]	Dental Follicle Stem Cells (DFSCs)	13–22, NS Teeth	*n* = 5	Evaluation of senescence through treatment with N-acetylcysteine (NAC)	SA-β-gal, p53, p21 ROS	mRNA (*Notch1*,* RUNX2*,* COL1*,* OCN*,* OPN*) ALP, ARS	Bone regeneration in extraction tooth sockets of NAC-treated mice (Allogeneic/ Local cell transplantation into extraction sockets)
Salamanna et al. [70]	Human bone marrow aspirate (BMA)	Mean age: 44 ± 0.5/ 68.6 ± 2.5, Female Clotted vertebral bone marrow aspirate	*n* = 3 per group	Evaluation of senescence and osteogenic capacity of clot vertebral BMA-derived MSCs from young and aged donors	SASP (IL-8, IL-1α, IL-1β, IL-6, CCL4L1, CXCL2, CCL2 TNFα)	mRNA (*COL1A1*,* RUNX2*,* OCN*,)	-
Gao et al. [29]	Mesenchymal Stromal Cells (MSCs)	NS, NS Adipose tissue	NS	The effect of GDF11 in MSC senescence caused by long-term culture	SA-β-gal p16 ROS	mRNA (*BMP2*,* RUNX2*,* IGF1*,* Tet2*) Protein (TET2, mTOR, AKT1) ARS	MicroCT in GDF11-treated mice (BV/TV, Tb.N, Tb.Sp, Tb.Th) -
Mullen et al. [25]	Adipose-Derived Stem Cells (ADSCs)	10–80, NS CellTex Therapeutics	*n* = 4	The effects of fisetin on culture-expanded senescence	SA-β-gal ROS SAHF (H3K9, γH2AX)	mRNA (*COL1*,* ALP*,* OC*,* PPARγ*,* C/EBPα*,* FABP4*) ARS, ORO	-
Zheng et al. [30]	Jawbone marrow MSCs (JBMMSCs)	60–75, NS Bone fragment	NS	Senescence due to diabetic osteoporosis (DOP)	SA-β-gal p53, p21, p16, SASP (TNF-α, IL-6, Mmp3)	mRNA (*RUNX2*,* BMP2*,* ALP*,* OCN*,* SP7*,* DLX5*) ALP, ARS	Bone regeneration in DOP rats (Xenogeneic/ Cell injection)
Liu et al. [36]	Bone Marrow-Derived MSCs (BMMSCs)	19–25, NS. Maxilla and iliac crest	*n* = 7	The effect of Phytic Acid (PA) on senescence due to hyperglycemia	SA-β-gal p53, p21 ROS	mRNA (*OPN*,* RUNX2*,* COL1*) Protein (OPN, RUNX2, COL1) ALP, ARS	Bone regeneration in extraction tooth sockets of PA-treated mice (Allogeneic/ Local cell transplantation into extraction sockets)
Zheng et al. [44]	Periodontal ligament stem cells (PDLSCs)	18–25, NS Third molar teeth	NS	The effect of Rapamycin on reversing H₂O₂-induced senescence and impaired osteogenic differentiation in PDLSCs	SA-β-gal p53, p21, p16	mRNA (*RUNX2*,* BSP*,* ALP*,* COL1*) Protein (COL1, RUNX2, AKT, PI3) ALP, ARS	Calvarial bone defects in rat treated by Rapamycin (Xenogeneic/ Local cell transplantation into bone defects)

### Senescence markers in bone regeneration

**Fig. 3:**
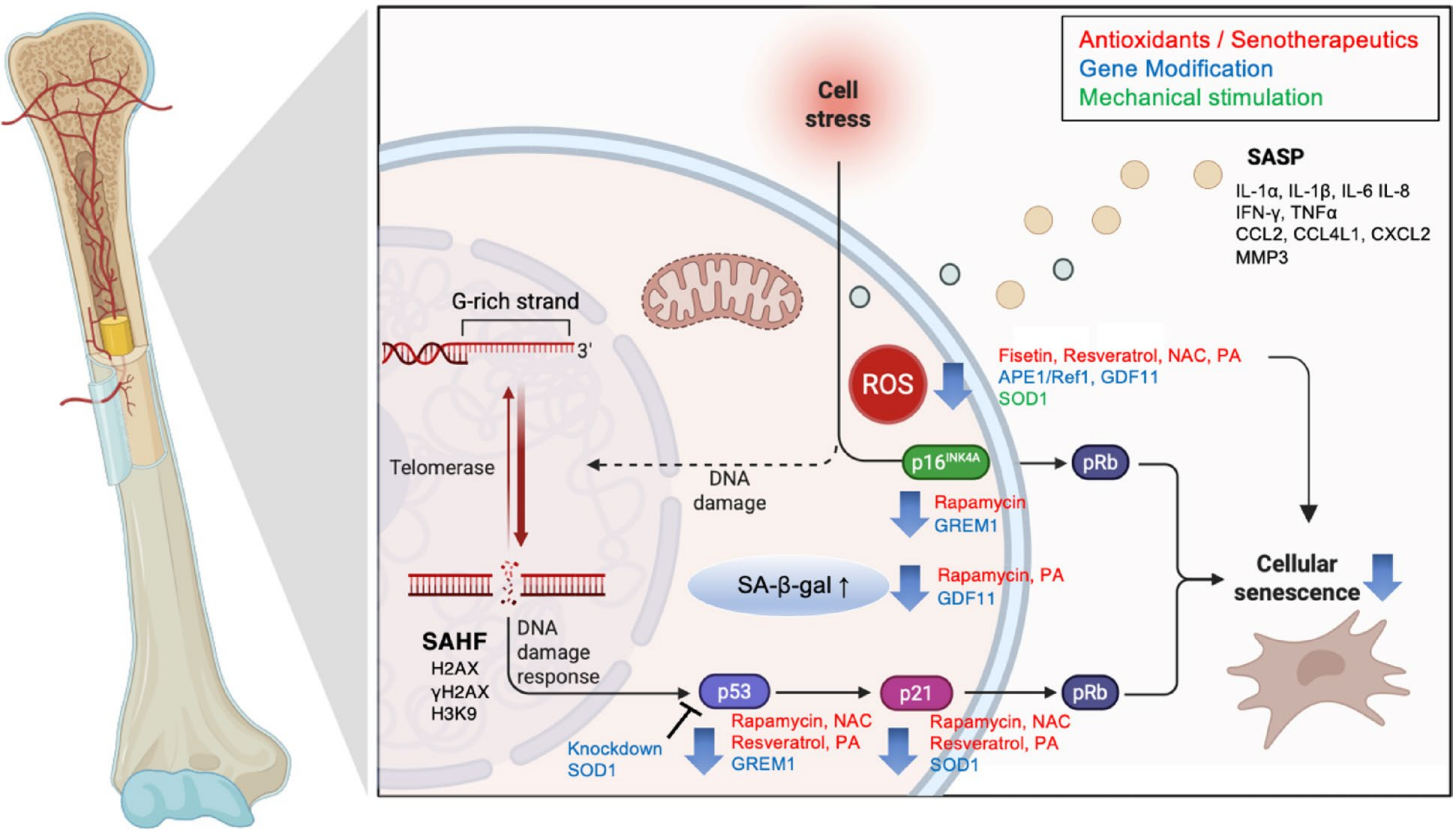
Senescence markers and their relationships

Due to the complex nature of aging, it is challenging to evaluate senescence using a single marker. Instead, a combination of indicators is necessary [21]. These include changes in cell size and morphology, increased β-Gal activity, elevated ROS levels, and the presence of senescence-associated secretory phenotype (SASP) components. Additionally, distinct senescence markers such as p16Ink4a, p21, and p53 also play a critical role in identifying cellular senescence and the overall aging process of the organism. These factors influence each other, creating an intricate network that contributes to senescence. Here, we summarize the senescence markers discussed in the 13 included studies. Important senescence markers and their relationships are summarized in Fig. 3.

####  SA-β-Gal

Senescence-associated β-galactosidase (SA-β-gal) is a lysosomal enzyme that hydrolyzes terminal β-D-galactose residues and serves as a widely accepted marker of cellular senescence [21, 22]. SA-β-gal expression is particularly prominent under conditions such as DNA damage and chemotherapy exposure, and has been associated with various age-related diseases including cancer and chronic inflammatory conditions. This makes it a relevant marker in the study of age-related changes in bone metabolism, where senescence impacts both osteogenic and osteoclastic activity [23]. While senescent cells show high SA-β-gal activity, this marker remains undetectable in quiescent or fully differentiated cells. However, SA-β-gal activity alone does not specifically identify senescent cells and is often evaluated in combination with other senescence markers.

Seven out of 13 papers investigated the expression of SA-β-gal, highlighting its prominence as a senescence marker across different cell types and experimental conditions. Among these studies, Gresham et al. examined senescence induction methods in mesenchymal stromal cells (MSCs) and reported that treatments with etoposide, hydrogen peroxide, and irradiation effectively increased SA-β-gal staining [24]. Mullen et al. showed that adipose-derived stem cells (ADSCs) exhibited increased SA-β-gal positivity during culture expansion, indicating senescence progression [25]. Similarly, Heo et al., in human bone marrow-derived mesenchymal stem cells (hBMSCs), SA-β-gal staining progressively increased with passage number, particularly pronounced at passages 9 and 11. SA-β-gal is commonly used to evaluate the therapeutic effects on cellular senescence [26]. Liu et al. demonstrated that Gremlin 1 (GREM1) overexpression in adipose-derived stem cells (ADSCs) significantly reduced the proportion of SA-β-gal-positive cells and suppressed the expression of senescence markers, including p16 and p53 [27]. Meng et al. reported that N-acetylcysteine (NAC) treatment reduced SA-β-gal staining in dental follicle stem cells (DFSCs) [28]. Gao et al. showed that administration of GDF11 in aged mesenchymal stem cells (MSCs) decreased SA-β-gal positivity and p16 expression, highlighting its potential anti-senescence effect [29]. Zheng et al. found that marrow MSCs derived from the jaw bones of diabetic osteoporosis patients exhibited markedly increased SA-β-gal positivity and elevated p53 expression [30].

Together, these findings suggest that SA-β-gal expression is elevated by factors such as prolonged culture, oxidative stress, induced pathological conditions. These conditions are widely used to assess various therapeutic effects on cellular senescence.

#### p53

p53 is a tumor suppressor protein that plays a crucial role in the cellular response to DNA damage and cellular stress. p53 plays a critical role in maintaining cellular integrity by inhibiting abnormal cell proliferation. In response to DNA damage, p53 temporarily halts the cell cycle to allow for repair processes to occur. If the damage cannot be repaired, p53 triggers apoptosis to remove the affected cells, thereby acting as a key mechanism in tumor suppression. Beyond its cancer-preventive functions, p53 is also integral to cellular senescence, where it arrests cell division and facilitates the development of senescence-associated secretory phenotype (SASP); studies have demonstrated that the p53-β variant can regulate cellular senescence [31].

Two studies suggested the potential of targeting p53 as a therapeutic strategy to prevent cellular senescence and promote bone regeneration. One study focused on preventing oxidative stress-induced cellular aging in dental pulp stem cells (DPSCs). In this study, superoxide dismutase (SOD1) was conjugated with low molecular weight protamine (LMWP) to enhance cellular uptake. This approach successfully suppressed the activities of p53 and p21, thereby reducing cellular aging in DPSCs [32].

Another study investigated the role of p53 in osteogenesis using MSCs derived from the jawbone of diabetic osteoporotic patients. Human jawbone marrow MSCs (hJBMMCs) obtained from patients with diabetic osteoporosis exhibited cellular senescence and reduced osteogenesis capacity. The study identified elevated p53 levels as the cause of this decreased osteogenesis ability. Additionally, overexpression of p53 was associated with increased expression of p16Ink4a and p21. There was also an increase in ROS production, leading to elevated levels of TNF-α, IL-6, and MMP3. Inhibiting p53 increased the expression of osteogenesis markers, thereby promoting bone regeneration, while overexpressing p53 suppressed these markers and hindered osteogenesis [30].

In both studies, p53 inhibition through the reduction of oxidative stress in DPSCs or by directly targeting p53 in hJBMMCs were shown to demonstrate improvements in cellular function and bone regeneration. The osteogenic potential of stem cells were shown to be enhanced by direct suppression of p53, that reduced cellular senescence and enhanced the expression of key osteogenic markers.

#### p21

p21 is a cyclin-dependent kinase inhibitor (CDKI) protein that primarily functions to induce G1/S or G2/M cell cycle arrest by the inhibition of cyclin-CDK complexes. This action prevents cells from proliferating, which is a hallmark of senescent cells. The expression of p21 is typically transient, showing an early increase followed by a significant decrease [33]. p21 has been linked to the regulation of SASP, which includes the secretion of pro-inflammatory cytokines, chemokines, and proteases that can influence the tissue microenvironment. The interplay between p21 and p16Ink4a in regulating SASP and maintaining the senescent state highlights their complementary roles in modulating the tissue microenvironment and influencing age-related pathologies [34]. Therefore, although it is known as a factor that promotes senescence, it has also been reported to play a role in eliminating senescent cells by reprogramming the surrounding environment to first recruiting macrophages and then inducing factors that attract immune cells [35].

The expression of p21 has been shown to significantly increase in response to DNA damage that leads to cell cycle arrest as a mechanism for managing cellular stress and ultimately contributing to the progression of cellular senescence [24, 26, 28]. Heo et al. and Choi et al. have both demonstrated that p21 expression is related to the activation of p53. p53 plays a central role in the cellular stress response with p21 reacting downstream to induce cell cycle arrest, combined to form part of the mechanism by which stem cells progress towards senescence [26, 32].

Liu et al. and Gresham et al. have both suggested the suppression of p21 expression can mitigate stem cell senescence and enhance regenerative capacity. Antioxidants such as phytic acid and fisetin were found to decrease p21 expression and maintain the regenerative potential of stem cells [24, 36]. Moreover, Liu et al. reported that suppression of p21 expression is linked to the activation of signaling pathways such as ERK and PI3K/AKT, which furthers p21 suppression and stem cell rejuvenation. Activation of these pathways has been shown to promote p21 expression [36].

These findings indicate that p21 is a key regulator in promoting cell senescence.

#### p16Ink4a

p16Ink4a is a protein that promotes cellular senescence by stopping the cell cycle and is specifically expressed in senescence cells. p16Ink4a inhibits enzymes that drive the cell cycle and activates the retinoblastoma protein (pRB) [37]. p16Ink4a, unlike other senescence markers such as p21 and p53, uniquely induces irreversible cellular senescence through cell cycle arrest. Specifically, p16Ink4a inhibits cyclin-dependent kinases (CDK) 4/6, leading to the activation of the retinoblastoma protein (pRB). This activation blocks the transition from the G1 phase to the S phase, halting cell proliferation and driving cells into senescence.

Four of 13 papers investigated the expression of p16Ink4a. Gresham et al. evaluated methods for inducing senescence in MSCs with p16Ink4a expression included as one of the assessment markers. The experiments showed that senescence-inducing agents such as hydrogen peroxide, etoposide, and radiation increased p16 expression and significant increases in other senescence markers particularly in cells treated with radiation and etoposide [24]. Zheng et al. reported that in the context of diabetic osteoporosis (DOP), the expression of senescence markers, including p53, p21 and p16 in MSCs are significantly increased compared to control MSCs that is associated with a decline in bone formation capacity [30].

Additionally, Gao et al. reported that Growth Differentiation Factor 11 (GDF11) phosphorylates SMAD2/3, activating the PI3K-AKT-mTOR pathway. The activation of this pathway increases the expression of Ten-Eleven Translocation Methylcytosine Dioxygenase 2 (TET2), resulting in an autoregulatory loop that enhances the expression of GDF11 itself. Activation of this pathway decreased the expression of the cellular senescence marker of p16Ink4a and promoted osteogenesis thereby emphasizing the importance of p16Ink4a in bone regeneration [29]. Liu et al. highlight that the suppression of p16 and expression is crucial to the anti-senescent effects of GREM1, suggesting that GREM1-mediated inhibition of p16 and p53 promotes the rejuvenation of ADSCs and enhances bone regeneration [27].

These findings indicate that p16 serves as a crucial senescence marker across various senescence-inducing conditions and under the influence of inhibitory agents.

#### Mitochondrial dysfunction and ROS

Mitochondrial dynamics is regulated by the processes of fusion and fission that play a critical role in cellular quality control to remove damaged mitochondria and prevent senescence and aging. As mitochondria age, their energy production capacity decreases, resulting in diminished cellular metabolic function. This leads to a reduction in regeneration and repair abilities, accelerating tissue aging. Additionally, mitochondria produce ROS during energy production. When ROS accumulate, they cause damage to cellular components that further increases the risk of age-related diseases due to elevated oxidative stress and overall ageing process. ROS are generated through various extracellular and intracellular actions including mitochondrial oxidative metabolism that can damage cellular components if not adequately neutralized by enzymes like superoxide dismutase (SOD), catalase, and glutathione peroxidase enzymes [38, 39]. Mitochondrial dysfunction is therefore an important source of elevated oxidative stress resulting in increased levels of ROScontributing to cellular damage and the senescence process [40].

Marycz et al. studied the effects of a colloidal suspension of nanocrystalline hydroxyapatite (nHP) loaded with resveratrol on the viability, metabolic activity, and mitochondrial function of human adipose-derived mesenchymal stromal cells (hASCs). nHP is a biocompatible material widely used in bone regeneration and tissue engineering, known for mimicking the main component of natural bone with a large surface area and high bioactivity. The hASCs were cultured with these hydrogels, and their proliferation, metabolic activity, mitochondrial function, and oxidative stress markers were evaluated. Hydrogels functionalized with nHP and resveratrol significantly improved the viability and proliferation of hASCs, with the 0.1 mM resveratrol/nHP hydrogel showing the most pronounced effects. Additionally, these hydrogels enhanced the metabolic activity and mitochondrial function of hASCs. The significant reduction in ROS levels, along with the downregulation of p21 and p53, indicated that these hydrogels possess strong antioxidative properties, improving the overall regenerative potential of hASCs [41].

These findings indicate that mitochondrial function and ROS production are highly indicative of cellular senescence that are associated with the proliferative and regenerative potentials of progenitor cells.

### Senotherapeutics

**Fig. 4:**
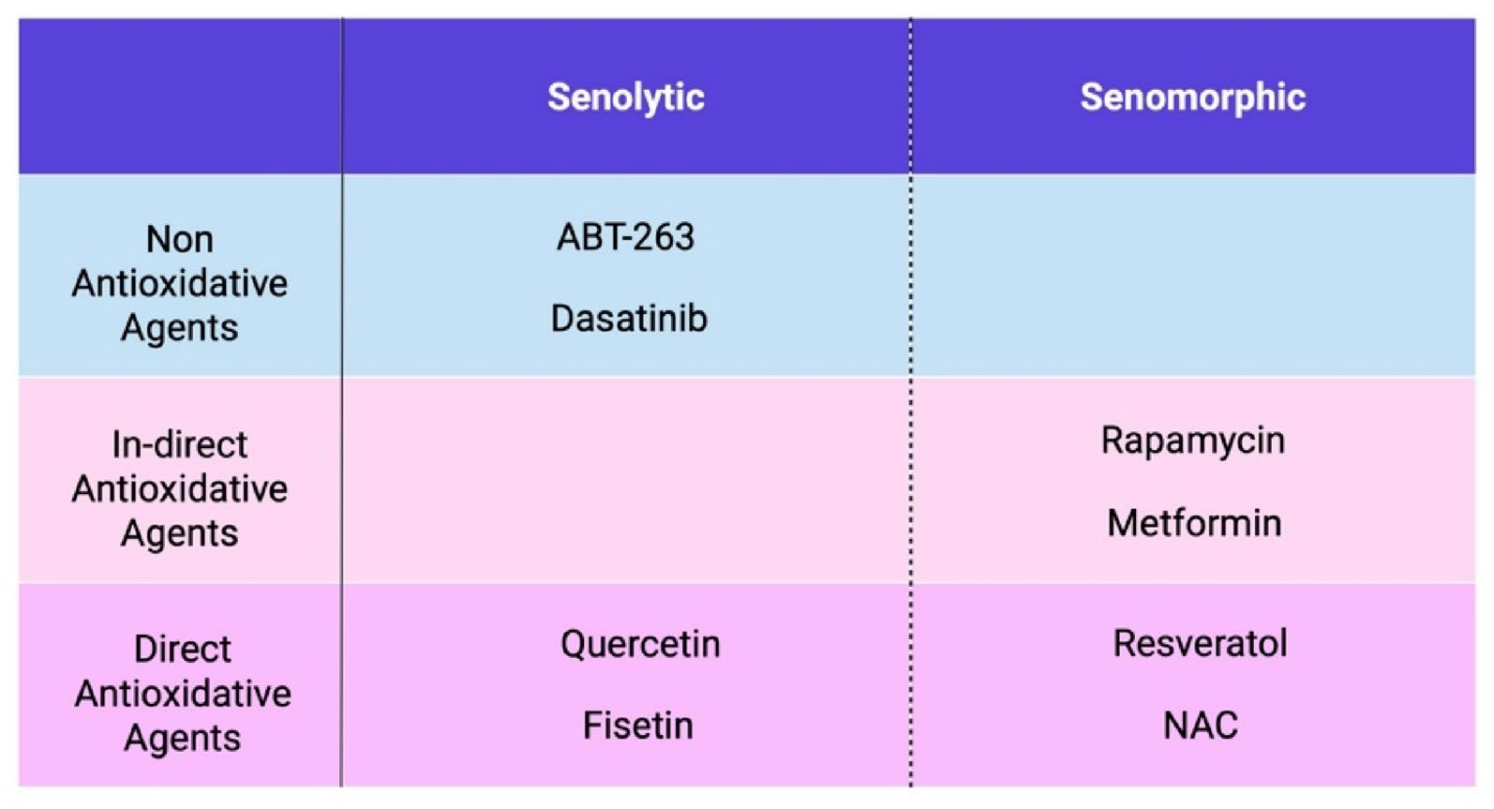
Therapeutic Strategies Targeting Cellular Senescence: A Classification of Senotherapeutics

Therapeutic strategies for senescence in bone regeneration from 12 studies are shown in Table 3 (Salamanna et al. (2022) was excluded due to the absence of therapeutic intervention). Aging inhibitors, also known as senotherapeutics, are to delay or reverse biological processes associated with senescence. These agents target key pathways involved in senescence, such as oxidative stress, inflammation, cellular senescence, and mitochondrial dysfunction [42]. They can be divided into two main types according to their mechanism of action: senolytics, which specifically eliminate senescent cells; and senomorphics, which suppress the harmful external effects exerted by senescent cells, with representative agents for each category summarized in Fig. 4.

**Table 3:** Summary of the major findings and conclusions in terms of biological pathways, treatment and effects from studies included in this systematic review. Abbrev: NS: not-specified

Treatment Category		Cell source	Aging Model	Treatment	Markers Studied/Relevant Changes in Treatment Group Relative to Positive Control (Biological Pathways)	Studies
Antioxidant & Senotherapeutics treatment	Senolytic: Flavonoid antioxidant	MSCs, Bone marrow derived cells (BMDCs)	X-ray irradiation	Fisetin	• MSCs: no change in DNA content, metabolic activity, p16 and p21 expression, intracellular ALP activity, and calcium deposition • BMDCs: reduced cellularity, lower expression of p16 and p21, reduced secretion of IL-6, increased metabolic activity, increased ALP activity, increased calcium deposition • (NS)	Gresham et al. [24]
	Senolytic: Flavonoid antioxidant	Adipose-derived stem cells (ADSCs)	Passaging	Fisetin	• Fisetin decreased proportion of SA-β-galactosidase positive cells • Fisetin decreased number of cells positive for M3K9me3 and γ-H2AX senescence-associated heterochromatin foci • No reduction in osteogenic capacity with fisetin treatment • (NS)	Mullen et al. [25]
	Senomorphic	Periodontal ligament stem cells (PDLSCs)	H₂O₂	Rapamycin	• Rapamycin treatment decreased proportion of SA-β-galactosidase positive cells • Rapamycin treatment reduced the expression of p16, p21, and p53, and enhanced metabolic activity, ALP activity, and bone mineralization (ARS) in PDLSCs • Rapamycin treatment enhanced bone regeneration, as indicated by increased bone volume and bone mineral density (BV/BMD), as well as a higher proportion of RUNX2-positive cells (IHC) in the calvarial defect model. • (PI3K/AKT signaling pathway)	Zheng et al. [44]
	Senomorphic Polyphenol: non-flavonoid antioxidant	Adipose-derived mesenchymal stromal cells (ADSCs)	NS	Resveratrol	• Resveratrol carried by nHAp (RES/nHAp) improved cell proliferation, mitochondrial activity, • RES/nHAp reduced intracellular ROS levels • RES/nHAp reduced the expression of apoptotic genes, including p21, p53, Bax, and increased the expression of the anti-apoptotic Bcl-2 and surviving • (Focused on mitochondria)	Marycz et al. [41]
	Senomorphic Amino acid derivative Non-flavonoid antioxidant	Dental follicle stem cells (DFSCs)	ROS	N-acetylcysteine	• NAC-treated hDFSCs had improved proliferation, lower senescence, higher stem cell-specific markers (CD44, CD90, Notch-1), higher levels of osteogenic factors (RUNX2, COL1, OCN, OPN), and increased calcium deposition • NAC treatment reduced ROS levels, induced antioxidant levels (glutathione, catalase, superoxide dismutase), and upregulated PI3K/AKT signaling • NAC treatment upregulated expression of anti-inflammatory cytokines/receptors (IL-4r, IL-16, TGF-β3, TGF-β2r, TGF-β3r, TGF-βi) and downregulated pro-inflammatory cytokines/receptors (IL-6r, IL-18) • (PI3K/AKT signaling pathway)	Meng et al. [28]
	Stretch-induced antioxidant responses (mechanical stretch)	Bone marrow-derived MSCs (BMMSCs)	ROS	Cyclic mechanical stretch	• Mechanical stretch decreased intracellular reactive oxygen species (ROS) by upregulating expression of SOD1 • Mechanical stretch increased expression of BGLAP, RUNX2, SP7, and ALP in BM-MSCs • Mechanical stretch increased bone matrix mineralization (calcium deposition) • (AMPK-SIRT1 signaling pathway)	Chen et al. [31]
Gene & Enzyme Therapy	Lentiviral-induced gene modulation	Mesenchymal stromal cells (MSCs)	NS	GREM1 shRNA	• Inhibition and/or knockdown of GREM1 expression increased osteogenic differentiation capacity, increased ALP activity, increased calcium mineralization, and increased OCN, OPN, DSPP, DMP1, BSP, OSX, and RUNX2 • Overexpression of GREM1 decreased proportion of SA-β-galactosidase positive cells, up-regulated telomerase activity, downregulated p16 and p53 • (Disrupting GREM1’s inhibition of BMP signaling pathway)	Liu et al. [27]
	Lentiviral-induced gene modulation	Mesenchymal stromal cells (MSCs)	Highly passaged cells	rGDF11	• Through the GDF11-PI3K/mTOR-Tet2-GDF11 autoregulatory loop, GDF11 increased expression of BMP2, RUNX2, and IGF1 • GDF11 increased calcium deposition, and modulated P16ink4a and telomere length • (SMAD2/3-PI3K-AKT-mTOR signaling pathway)	Gao et al. [29]
	Adenoviral- induced gene modulation	Bone marrow-derived MSCs (BMMSCs)	H₂O₂	AdRef-1	• Suppressed intracellular superoxide levels • Decreased proportion of SA-β-galactosidase positive cells • (APE1/Ref-1)	Heo et al. [26]
	Gene knockdown therapy	Jawbone marrow MSCs (JBMMSCs)	ROS	p53 siRNA	• p53 siRNA knockdown decreased p53, p21, and p16 expression, and increased mineralization and expression of RUNX2, ALP, and DLX5 • (p53 signaling pathway)	Zheng et al. [30]
	Enzyme therapy with cell-penetrating peptide	Dental pulp stem cells (DPSCs)	H₂O₂	LMWP-SOD1	• Restoration of natural DPSC morphology • Significant reduction of SA-β-galactosidase activity • Decreased number of cells in G1 phase • Abolition of increased p53 and p21Cip1/WAF1 expression • Significant reduction of alkaline phosphatase, type I collagen, osteopontin, and runx2 expression • (p53-p21Cip1/WAF1 pathway)	Choi et al. [32]
Pharmacotherapy	Organic phosphoric acid-based treatment	Bone marrow-derived MSCs (BMMSCs)	Senescence due to hyperglycaemia	Phytic acid	• Phytic acid promoted hBMSC proliferation, increased ALP activity, increased calcium deposition, and increased expression of ALP, OPN, RUNX2, and COL-1 that otherwise decreased in a high-glucose environment • Phytic acid decreased inflammatory factors (TNF-α, IL-1 β) and inhibited activation of TLR4-NFκB and Nrf2 inflammatory pathways • (MAPK signaling pathway (JNK, ERK, P38); TLR4-NFκB and Nrf2 pathways)	Liu et al. [36]

*Salamanna et al. (2022) was excluded due to the absence of therapeutic intervention

Two of the 13 papers examined the effects of fisetin, a senolytic, on bone regeneration. Fisetin is a naturally occurring flavonoid with antioxidant and senolytic properties. Fisetin reduces the accumulation of senescent cells by targeting anti-apoptotic pathways, particularly by inhibiting BCL-2 family proteins, thereby selectively inducing apoptosis in senescent cells. Gresham et al. evaluated the senolytic effects of fisetin in a composite system of senescent and non-senescent MSCs encapsulated within GelMA hydrogels. Although fisetin treatment did not markedly reduce senescence markers or enhance osteogenic potential in MSC-only cultures, its application in a heterogeneous cell environment, incorporating BMDCs, resulted in a significant decrease in senescence markers and an increase in osteogenic differentiation. This finding underscores fisetin’s efficacy within mixed-cell populations, where it effectively reduces cellular senescence and supports bone regenerative potential [24]. Mullen et al. investigated fisetin’s impact on the accumulation of senescence markers in ADSCs during in vitro culture expansion. Fisetin demonstrated a dose-dependent reduction in senescence-associated markers, including ROS, β-galactosidase activity, and SAHF (senescence-associated heterochromatin foci). By selectively clearing senescent cells, fisetin preserved ADSC proliferation and differentiation capabilities, positioning it as a promising anti-senescent agent for regenerative applications using ADSCs [25].

One of the 13 papers investigated a senomorphic. Zheng et al. investigated the effect of rapamycin on reversing H₂O₂-induced senescence and the associated impairment of osteogenic differentiation in periodontal ligament stem cells (PDLSCs). Rapamycin is a macrolide compound isolated from the bacterium *Streptomyces hygroscopicus*. Originally an anti-fungal agent, rapamycin is now recognized as a well-established senomorphic that attenuates cellular senescence and suppresses SASP expression across multiple species and senescence models. Previous studies have demonstrated that rapamycin exerts its senomorphic and longevity-promoting effects primarily through inhibition of the mTOR pathway, especially via TORC1 suppression, leading to reduced phosphorylation of S6K and 4E-BP, activation of the Nrf2 pathway, and attenuation of NF-κB–mediated pro-inflammatory signaling [43]. In this study, Zheng et al. reported that rapamycin promoted osteogenic differentiation of PDLSCs via the PI3K/AKT signaling pathway in vitro, as evidenced by the. increased AKT and PI3K phosphorylation in the rapamycin-treated group. Moreover, experiments using the PI3K/AKT inhibitor LY294002 demonstrated that inhibition of this pathway attenuated the rapamycin-induced upregulation of RUNX2 and COL-1 expression as well as the promotion of osteogenic differentiation. Furthermore, in the calvarial bone defect model, rapamycin was shown to restore the function of senescent cells induced by H₂O₂, suggesting its potential therapeutic effects on bone regeneration in the context of senescence [44].

## Discussion

This systematic review highlights the effects of cellular senescence in impairing the regenerative processes of bone. It is important to recognize the considerable variability in MSCs based on their anatomical source, culture conditions, donor characteristics, and methods of cell isolation. This heterogeneity can significantly impact functional outcomes, such as differentiation capacity, immunomodulatory properties, and anti-inflammatory effects, which may lead to inconsistencies in therapeutic efficacy [45]. Moreover, the variability in physiological environments across different age groups requires further investigation to ensure that treatment strategies are effective across a broad population.

Cellular senescence in MSCs significantly reduces their ability for osteogenesis. Senescent MSCs lose their typical fibroblast-like shape and adopt a flatter, elongated morphology, with a notable increase in size and granularity [46–48]. Using human cells, four methods of inducing senescence (replicative senescence, H_2_O_2_ treatment, etoposide treatment, and irradiation) were compared, with irradiation proving to be the most effective [24]. The study showed that senescent MSCs exhibit increased inflammation and altered differentiation, being more prone to adipogenesis than osteogenesis. Senescent MSCs exhibit increased expression of senescence markers such as p16Ink4a, p21, and p53, which contribute to the arrest of cell growth and diminished osteogenic capacity [25, 27, 28, 30, 32, 36, 37, 49]. Additionally, SASP creates a local inflammatory microenvironment that hinders bone regeneration. The accumulation of senescent cells leads to the development of SASP. This promotes inflammation (including increased levels of IL-1β, IL-6 and TNFα), protein degradation, and tissue dysfunction, further inducing senescence in nearby cells and tissues [50]. Additionally, SASP impairs the maintenance, regeneration, and repair of musculoskeletal tissues [51–53]. In summary, it is generally reported that cellular senescence characterized by the increase in senescence markers expression is associated with decreased osteogenic capacity of MSCs.

Out of 13 studies, two examined SASP factors. Gresham et al. observed that senescent MSCs exhibit elevated levels of IL-1β, IL-6, and IFN-γ, particularly in stress-induced senescence models like etoposide and irradiation [24]. Zheng et al. demonstrated that TNFα, MMP-3, and MMP-13 were significantly increased in the diabetic osteoporosis (DOP) group, where the osteogenic ability of hJBMMSCs was notably reduced in the DOP environment [30]. These SASP factors were shown to promote chronic inflammation, enhance osteoclast activity, and disrupt the bone matrix, ultimately impairing bone healing and regeneration [54, 55].

By understanding the biology of senescence markers in MSCs, potential strategies directly targeting these markers to modulate cellular senescence may be developed to favorably impact clinical outcomes. Out of the 13 papers, 5 focused on gene modification as a therapeutic approach. When p53, p21, and p16 expression levels are elevated, cells are prone to cell cycle arrest, senescence, or programmed cell apoptosis. The accumulation of senescent cells expressing high levels of p21 or p16 contributes to a pro-inflammatory environment, disrupting tissue homeostasis and impairing functions essential for processes like osteogenesis and other reparative mechanisms. This chronic inflammatory environment exacerbates tissue dysfunction, hindering effective regeneration and repair. Therefore, current literature generally supports the theory that cellular senescence characterized by elevated expression of the above senescence markers is associated with a chronic low level inflammatory microenvironment.

Among these markers, p53 plays a pivotal role in the regulation of cellular senescence, apoptosis, and the broader senescence process. However, the cellular context such as the tissue or cell type, the cell’s proliferative capacity, and the nature of the damage, determine whether p53 induces senescence or apoptosis. In cases of severe damage or stress, p53 triggers apoptosis via factors such as Bax and PUMA [56, 57] However, in response to mild DNA damage or stress, p53 induces cell cycle arrest, which is associated with the increased expression of other senescence markers, such as p16Ink4a and p21. This ultimately leads to the cessation of cell growth and the onset of senescence. As a senescence marker, p53 activation triggers the production of reactive oxygen species (ROS), contributing to cellular stress and the induction of the SASP, as well as matrix metalloproteinases such as MMP3. These factors contribute to the arrest of cell growth and the overall decline in regenerative potential, including diminished osteogenesis. In senescent cells, the role of p53 is crucial in regulating the cellular response to stress and damage, and it plays a significant part in the overall senescence process. This includes influencing the decline in osteogenesis capacity and limiting the regenerative potential of tissues. Thus, targeting p53 is a potential therapeutic strategy to prevent cellular senescence and promote bone regeneration. The studies referenced in this paper suggest that while p53 knockdown and inhibition of p53 via regulation of the p53-p21 pathway can enhance osteogenic differentiation and reduce cellular senescence, potential drawbacks may arise due to the broader regulatory roles of p53 in cellular homeostasis. As a critical tumor suppressor, downregulation or mutation of p53 may impair the cell’s ability to arrest the cell cycle in response to DNA damage, thereby potentially increasing the risk of uncontrolled cell proliferation or malignancy. However, it has been reported that loss or inactivation of p53 alone is insufficient to initiate tumorigenesis; while it facilitates the development of a pro-tumorigenic environment, additional genetic or environmental insults are typically required to drive malignant transformation [58, 59]. Furthermore, p16 and p21, which are downstream effectors of p53 and traditionally recognized as tumor suppressors, have also been implicated in tumor-promoting activities under specific conditions, particularly through enhancing the chemotactic activity of monocytic myeloid-derived suppressor cells (Mo-MDSCs). These CDK inhibitors upregulate CX3CR1 expression via SMAD3 signaling, thereby promoting Mo-MDSC infiltration into tumors and facilitating immune evasion and tumor progression [60]. This paradoxical role highlights the complexity of their functions in cancer biology and underscores the need for further investigation to elucidate their context-dependent effects on tumorigenesis. Zheng et al. emphasize the need for careful management of p53’s effects on cell differentiation and proliferation. Similarly, in Choi et al.‘s study, while inhibition of the p53-p21 pathway via superoxide dismutase (SOD1) conjugates showed efficacy against oxidative stress-induced senescence, the effects of p53 inhibition on cellular stress responses and long-term stability have not been fully explored. Thus, there is an indication that complete p53 suppression may not be optimal, warranting caution in clinical applications. The expression of p16Ink4a is another hallmark of senescent cells with ongoing research into targeting these cells for selective removal aiming to rejuvenate tissues [61, 62]. This approach holds the potential to prevent tissue dysfunction and slow the progression of age-related diseases caused by the accumulation of senescent cells. In summary, our review has identified that these most common senescence markers being proposed as direct interventional targets to manipulate the progression of cellular senescence. However, many of the primary studies have missed to incorporate sampling from different age groups thus dampening the power of the studies.

In our review, we have also identified other senescence-related biological pathways that are commonly targeted to achieve the goal of bone regeneration.

It is well reported that MSCs undergo senescence with aging, leading to diminished regenerative capacity. In the context of bone regeneration, several cell source-related factors are associated with increased MSC senescence and impaired function. Advanced age is a primary factor contributing to cellular aging, which results in reduced proliferation, migration, and osteogenic differentiation potential of MSCs [63]. Additionally, disease states such as diabetes mellitus have been shown to exacerbate MSC dysfunction through mechanisms involving chronic inflammation, impaired angiogenesis, and metabolic dysregulation. Oxidative stress is another critical contributor, as elevated reactive oxygen species (ROS) levels promote DNA damage and senescence, further impairing the regenerative potential of MSCs. Thus, many of the anti-senescence interventions tend to target oxidative stress or ROS accumulation. Of the 13 studies included in this review, 7 reported that reducing ROS levels effectively attenuated cellular senescence. These findings underscore the pivotal role of oxidative stress in senescence and highlight the potential therapeutic benefit of targeting ROS reduction to mitigate age-related cellular dysfunction and enhance bone regeneration. ROS produced during mitochondrial energy metabolism contribute directly to oxidative stress, accelerating cellular dysfunction and the onset of senescence. Therefore, maintaining mitochondrial integrity is essential for sustaining bone regeneration. In this context, therapeutic strategies aimed at reducing oxidative stress and improving mitochondrial function offer a promising approach to counteracting the negative effects of cellular senescence and promoting bone regeneration.

Additionally, therapeutic strategies targeting cellular senescence known as senotherapeutics represent a highly active area of research in bone regeneration and tissue repair. Among the agents covered in this review, senolytic agents like fisetin have demonstrated efficacy in selectively eliminating senescent cells and enhancing cellular function, while other methods and agents are also being explored as promising candidates for applications in regenerative medicine. Preclinical studies have demonstrated that fisetin effectively reduces cellular senescence in aged C57BL/6 mice, extending the lifespan of 85-week-old mice by approximately 3 months and leading to improved health outcomes [64]. Based on these findings, clinical trials have been initiated to assess the efficacy of fisetin in elderly populations, with a focus on markers of frailty, inflammation, insulin resistance, and bone metabolism. In addition to fisetin, other senotherapeutic such as ABT-263, dasatinib, and quercetin as senolytics, and metformin, rapamycin, resveratrol, and NAC as senomorphics are expected to become key focuses of future research aimed at evaluating their potential clinical applications. In addition, the therapeutic effects of other senolytics on cells particularly the cocktail therapy of dasatinib and quercetin (D་Q) have recently attracted attention, with expectations for further therapeutic benefits [65]. D་Q has been reported to improve the osteogenic capacity of aged BMSCs in vitro and in vivo in mouse models [66], but studies on human BMSCs remain limited, and further investigation in this field is anticipated for bone regeneration.

Although the primary focus of this review was senescence markers, and therefore studies using human iPSCs were not directly included in our analysis, it is noteworthy that within the 101 studies categorized under the proportion of cell sources used as intervention strategies in bone regeneration, some reports investigated iPSC-based approaches. Among them, Spitzhorn et al. demonstrated that iPSCs reprogrammed from aged donors and differentiated into iPSC-derived MSCs (iMSCs) acquire a rejuvenation-associated gene signature, including INHBE, DNMT3B, POU5F1P1, CDKN1C, and GCNT2, and regain a fetal-like secretome. These changes enable iMSCs to circumvent age-related declines in osteogenic potential [67]. This study emphasized that the regenerative effects of MSCs are thought to rely largely on paracrine mechanisms mediated by secreted factors rather than directly by the cells themselves. In a complementary approach, Hanetseder et al. showed that extracellular matrices generated from iPSC-derived mesenchymal progenitors (iECM) significantly enhanced osteogenesis of bone marrow stromal cells from both young and aged donors. Strikingly, aged MSCs cultured on iECM exhibited restored mineralization capacity absent on standard culture substrates, highlighting the promise of iPSC-derived matrices to provide a rejuvenated microenvironment [68].

Reprogramming into iPSCs induces an epigenetic reset leading to cellular rejuvenation. However, only a limited number of studies have systematically investigated how senescence markers are altered during this process. Demonstrating changes in the expression of such markers would provide critical evidence for the reliability and safety of iPSC-based approaches in regenerative medicine. In the context of bone regeneration, verifying whether senescence markers are indeed reduced in iMSC- or iECM-based therapies is indispensable for assessing the durability of therapeutic effects, the risk of persistent SASP-driven inflammation, and the potential for tumorigenesis or abnormal differentiation. Moreover, as a bridge to anti-aging research, understanding the behavior of senescence markers is particularly important. While iPSC-based therapies are often challenged by concerns regarding tumorigenic risk and difficulties in controlling lineage-specific differentiation, incorporating the dynamics of senescence markers would help determine whether these therapies truly represent “anti-aging” interventions or merely reset the cellular phenotype of aging.

The primary aim of this systematic review was to identify senescence markers associated with bone regeneration. To minimize the risk of overlooking potentially relevant but less frequently investigated markers, we did not restrict our search to a predefined set. Although markers such as p19^ARF^ and H3K9me3 have been reported in the broader literature, no studies to date have examined their roles in bone regeneration [69].

Notably, the majority of included studies induced cellular senescence in vitro using experimental manipulations, rather than assessing cells derived from physiologically aged donors. While a limited number of studies utilized cells from elderly individuals, only one of the 13 studies directly compared aged and young cells, and this investigation was constrained by a small sample size. Salamanna et al. reported that the expression levels of SASP factors (IL1α, IL1β, IL-6, IL-8, TNFα, MCP-1, CCL4, and CXCL2) in MSCs derived from clotted vertebral bone marrow aspirate from younger and older donors showed no significant differences. They concluded that age-related exacerbation of SASP is unlikely to impair bone regenerative capacity, and that these cells retain sufficient potential for bone regeneration therapy [70]. These findings underscore the need for future large-scale studies employing physiologically aged human cells to more accurately elucidate the role of cellular senescence in bone regeneration.

With the progression of an aging society, elderly individuals represent the primary target population for regenerative therapies. However, age-related declines in the regenerative capacity of MSCs limit their effectiveness in bone regeneration. Thus, novel therapeutic strategies that counteract MSC senescence, such as senolytics or iMSC/iECM-based approaches, hold considerable clinical significance. In this context, elucidating senescence markers is particularly important, as they provide critical insights into treatment efficacy and safety. This review has highlighted that current studies on senescence markers in bone regeneration remain largely confined to the in vitro stage, with relatively few in vivo evaluations. To advance toward clinical application, future research should include validation studies in animal models, particularly aged models that incorporate senescence-associated factors, and explore combinatorial strategies with existing biomaterials.

The data for this review were exclusively sourced from PubMed, Web of Science, Embase and Scopus, potentially omitting relevant studies from other databases. Despite this potential shortcoming, a comprehensive understanding of the fundamental mechanisms of cell senescence in bone regeneration could provide new insights into improving regenerative therapies in our aging population. This systematic review presents several novel contributions to the field of stem cell research and therapy by targeting cellular senescence in progenitor cells. Unlike previous narrative reviews, it systematically and quantitatively evaluates the relationship between senescence markers and bone regeneration outcomes, with a unique focus on human-derived cell sources. The review is the first to map the distribution of these cell sources in senescence research, providing foundational data for future clinical translation. It identifies specific senescence markers (such as p16, p21, p53, SA-β-Gal, and ROS) as proposed therapeutic targets and critically appraises common study design flaws, particularly the lack of age- and disease-diverse samples. The paper clarifies the mechanisms of cell therapy, distinguishing paracrine effects from direct engraftment, and expands the discussion on the potential and challenges of iPSC-based therapies, including safety and standardization issues. We attempted to provide comprehensive tabulation of findings and a focused discussion on clinical translation and future research directions to offer a new perspective and roadmap for advancing the field.

## Data Availability

All data generated or analyzed during this study are included in this published article.

## Abbreviations

ADSCs: Adipose-derived stem cells
APE1/Ref1: Apurinic/apyrimidinic EndSIRT1onuclease 1/redox factor-1
BMDCs: Bone marrow-derived cells
CCL4: Chemokine (C-C Motif) ligand 4
CDKN1C: Cyclin dependent kinase inhibitor 1 C
COL-1: Collagen type 1
CXCL2: Chemokine (C-X-C Motif) ligand 2
DFSCs: Dental follicle stem cells
DPSCs: Dental pulp stem cells
DNMT3B: DNA methyltransferase 3 beta
ERK: Extracellular signal-regulated kinase
GCNT2: Glucosaminyl (N-Acetyl) transferase 2
GDF11: Growth differentiation factor 11
GERM1: Germ cell nuclear factor 1
hAFSCs: Human amniotic fluid stem cells
HAOBs: Human osteoblasts isolated from aged alveolar bone
hBMMSCs: Human bone marrow mesenchymal stromal cells
hfSDSCs: Human fetal synovium-derived stem cells
hJBMMCs: Human Jawbone Marrow Mesenchymal Stem cells
hPDLF: Human periodontal ligament fibroblasts
iECM: iPSC-derived mesenchymal progenitors
IFN-γ: Interferon gamma
IL-1α: Interleukin-1 alpha
IL-1β: Interleukin-1 beta
IL-6: Interleukin-6
IL-8: Interleukin-8
iPSC: induced pluripotent stem cell
iMSCs: iPSC-derived MSCs
INHBE: Inhibin beta E
LMWP: Low molecular weight protamine
MCP-1: Monocyte chemoattractant protein-1
MMP-13: Matrix metalloproteinase 13
MMP3: Matrix metalloproteinase 3
MSC: Mesenchymal stromal cell
NAC: N-Acetylcysteine
PA: Phytic acid
PI3K/AKT: Phosphoinositide 3-Kinase/Protein Kinase B
POU5F1P1: POU class 5 homeobox 1 pseudogene 1
PRISMA: Preferred reporting items of systematic reviews and meta-analyses
RES/nHAP: Resveratrol/nano-hydroxyapatite
ROS: Reactive oxygen species
RUNX2: Runt-related transcription factor 2
SA-β-Gal: Senescence-associated β-galactosidase
SAHF: Senescence-associated heterochromatin foci
SASP: Senescence-associated secretory phenotype
SIRT1: Sirtuin 1
SOD1: Superoxide dismutase 1
TET2: Ten-eleven translocation methylcytosine dioxygenase 2
TNFα: Tumor necrosis factor alpha
UC-MSCs: Umbilical cord-mesenchymal stromal cells
